# Towards effective restriction of unhealthy food marketing to children: unlocking the potential of artificial intelligence

**DOI:** 10.1186/s12966-023-01458-6

**Published:** 2023-05-26

**Authors:** Dana Lee Olstad, Emma Boyland

**Affiliations:** 1 Department of Community Health Sciences, Cumming School of Medicine, University of Calgary, 3280 Hospital Drive NW, Calgary, AB T2N 4Z6 Canada; 2 Department of Psychology, Institute of Population Health, University of Liverpool, Bedford Street South, Liverpool, L69 7ZA UK

**Keywords:** Unhealthy food marketing, Children, Policy, Chile, Artificial intelligence

## Abstract

The World Health Organization recommends that member states enact policies to limit unhealthy food marketing to children. Chile enacted relatively stringent laws that restrict unhealthy food marketing to children in two phases, beginning in 2016. Dillman-Carpentier and colleagues examined the incremental effectiveness of the first and second phases of Chile’s policy in limiting children’s exposure to unhealthy food marketing on television relative to pre-policy. Banning advertisements for all ‘high-in’ products (i.e., those that exceeded thresholds for energy, saturated fats, sugars and/or sodium) during the daytime (phase 2) was more effective in reducing children’s exposure to unhealthy food marketing on television than only banning ‘high-in’ marketing during programs with large child audiences (phase 1). These findings underscore the importance of implementing comprehensive policies that reduce children’s exposure to all marketing for unhealthy foods—not simply that which targets them directly—to better protect them from its negative impacts. However, although policies in Chile and other nations have reduced children’s exposure to unhealthy food marketing in broadcast media, it is not clear whether such policies have meaningfully reduced children’s overall food marketing exposures. This is partly due to the challenges of studying children’s digital food marketing exposures, which are an increasingly important source of unhealthy food marketing. To address these methodologic gaps, several research teams are developing artificial intelligence (AI)-enabled systems to assess food marketing to children on digital media and support efforts to monitor compliance with policies that restrict this marketing. These and other AI systems will be essential to comprehensively and systematically study and monitor food marketing to children on digital media internationally and at scale.

The World Health Organization (WHO) first recommended that member states develop strategies to address unhealthy food marketing to children in 2004 [1]. This early call to action was followed by dozens of reports and research studies by the WHO and others confirming that unhealthy food marketing is pervasive and influences children and youth to prefer, request and consume energy-dense and nutrient-poor foods and beverages [2]. Based on this large literature showing unequivocal evidence of harm, the WHO called on member nations to enact strong and comprehensive policies to restrict unhealthy food marketing to children in 2010 [3]. Action in this respect continues to be at the top of the WHO’s research and policy agenda.

Despite a clear consensus on the need for action, very few countries have implemented mandatory policies restricting unhealthy food marketing to children [4]. That so few countries have succeeded in restricting food marketing to children points to the success of large multinational firms in framing the issue as a matter of parental—not state—responsibility, thereby alleviating pressure on governments to regulate [5]. Moreover, most developed nations are democracies with capitalist economies in which corporate profitability and free speech considerations often take precedence over child health. Nevertheless, in recent years several nations have succeeded in enacting relatively strong laws governing aspects of food marketing to children [4, 6]. Chile’s policy is among the most stringent of these laws. Beginning in 2016 (phase 1), products high in energy, saturated fats, sugars and/or sodium (*hereafter ‘high-in’ products*) could not be advertised on children’s media or where children constituted > 20% of the audience [7]. Child-directed marketing strategies were also prohibited in advertisements for such products. The second phase of the regulations—implemented in 2018—strengthened the nutrient thresholds for defining ‘high-in’ advertising and prohibited all ‘high-in’ advertising on television between 6am and 10pm, and after 10pm if the ads were directed at children.

In a recent systematic review, Boyland et al. [6] found that polices to restrict food marketing to children can effectively reduce children’s exposure to food marketing and its persuasive power, and may also reduce their purchases of unhealthy foods. However, the evidence was too limited to draw any firm conclusions regarding the impact of policy on children’s dietary intake. With respect to Chile’s policy more specifically, evidence indicates that the first phase of Chile’s regulations was associated with reductions in children and youth’s exposure to unhealthy food marketing on television and with declines in high-in food consumption [8, 9]. However, the declines in food marketing did not mediate associations between policy exposure and food consumption, raising questions as to the underlying mechanisms of action.

The current paper by Dillman-Carpentier and colleagues [10] provides further evidence that Chile’s policy has meaningfully reduced children’s exposure to unhealthy food marketing on television. The authors analyzed a stratified random sample of two weeks of television recordings from Chile’s four main stations aired between 6am and 12am annually, encompassing the pre-policy period (2016), phase 1 (2017, 2018) and phase 2 (2019) of the regulations. Trained raters coded advertisements to identify child-targeting techniques and rated the healthfulness of marketed products according to the more stringent phase 2 nutritional criteria. Children’s marketing exposures were quantified using gross rating points—a standard metric that indicates the proportion of children in a given area who were likely reached by an advertisement. Findings indicated that children’s exposure to marketing of ‘high-in’ products on television was 57% lower after phase 1 and 73% lower after phase 2 relative to the pre-regulation period. Thus, banning all ‘high-in’ ads during the daytime (phase 2) was more effective in reducing children’s exposure to unhealthy food marketing than only banning ‘high-in’ marketing during programs with large child audiences (phase 1).

Dillman-Carpentier and colleagues’ findings are analogous to findings in the UK, where food marketing restrictions on television were also implemented across two phases. In phase 1 (from January 2008) advertisements for foods high in fats, sugar, and/or salt (HFSS) were no longer permitted in or around programs likely to be of particular appeal to children, and dedicated children’s channels were required to scale back their HFSS advertising to no more than 50% of 2005 levels. In phase 2 (from January 2010) total removal of all HFSS advertising from children’s channels was enforced. Evaluations after the first phase of implementation found reductions of 39% and 56% for advertising exposure and power (specifically use of celebrities in food advertisements) respectively, whereas after full implementation the reductions were 52% and 84% [11]. Taken together, findings in Chile and the UK underscore the importance of enacting comprehensive policies that reduce children’s exposure to all marketing for unhealthy foods—not simply that which targets them directly—to better protect them from its negative impacts.

Although policies in Chile and other nations have reduced children’s exposure to unhealthy food marketing on broadcast media, it is not clear whether such policies have meaningfully reduced children’s overall food marketing exposures. This is partly because industry is increasingly leveraging the power of digital media to push advertisements for unhealthy foods to children in real-time, often using artificial intelligence (AI)-enabled techniques [12]. As many aspects of children’s lives are mediated by digital media, food marketing has now become embedded within virtually every aspect of children’s lives—from their leisure to their entertainment, school work and social relationships [12]. Digital food marketing is also frequently integrated with content, making it more difficult for children to discern its commercial intent and initiate consumer defenses [13–15]. The adverse impacts of unhealthy food marketing on digital media may therefore be more harmful to children than more traditional broadcast marketing.

It is challenging to study children’s exposure to digital food marketing and its impact on their dietary patterns, however. This is in part due to methodologic constraints. One particular challenge is that industry has leveraged AI and other technology-enabled strategies to market unhealthy foods to children on digital media, while researchers—particularly those with limited resources—continue to rely primarily on manual methods to assess the extent and power of this marketing (e.g., by visiting relevant digital platforms and counting food marketing instances). Although useful, such approaches cannot fully engage with the massive volume, varied and dynamic nature of digital food marketing. Reliance on manual methods also makes it challenging to monitor adherence to policy that restricts digital food marketing at scale, which is crucial to ensure policy effectiveness.

To address these methodologic gaps, several research teams are developing AI-enabled systems to assess food marketing to children, including on digital media, and to support efforts to monitor compliance with policy that restricts this marketing. For instance, a team based in the UK have developed a deep learning workflow to automatically extract and classify advertisements from still images [16]. Researchers in Australia are also developing machine learning algorithms within an image recognition system that automatically identifies and classifies instances of unhealthy food marketing within both digital and non-digital environments [17]. In Canada, researchers are developing an AI system that continuously monitors food marketing to children on websites, social media platforms, YouTube videos and gaming apps [18]. For each marketing instance, the AI system extracts marketing features to determine whether foods are present, classifies their healthfulness using Health Canada standards, and categorizes marketing strategies that target children. The system can be used to study children’s short- and long-term exposure to food marketing on digital media in a comprehensive and systematic manner and can monitor adherence to policies that restrict digital food marketing. It can also be used to identify food marketing within screen capture videos provided by children, thereby capturing personalized food marketing that children view on websites, social media, and others.

The advantages of using AI to assess food marketing to children include its scalability, reproducibility, consistency, and ability to capture marketing embedded in audio, text, still images, videos, immersive virtual reality experiences and others. However, AI-enabled approaches have limitations as AI systems must be trained on large volumes of advertisements—ideally labelled by children themselves—to identify food marketing that targets children. It is challenging to train AI systems to identify when music, language, colours, shapes, font styles and other marketing techniques explicitly target children given the vast diversity of advertisements and combinations of marketing strategies they employ. This is an important reason why the current findings are so noteworthy. Were other countries to follow Chile’s lead and prohibit all ‘high-in’ food marketing in specific contexts (e.g., no ‘high-in ads’ in certain settings, times of day), and to additionally extend such regulations to digital media (e.g., no ‘high-in’ ads on certain websites), there would be no need to train AI systems to identify child-targeting techniques. Such regulations would have the additional advantage of reducing adults’ exposure to unhealthy food advertisements, which may improve their diet quality and health over the longer term. Notably, however, regulating digital media is extremely challenging because digital media are borderless, and thus international cooperation is required [12].

AI will be essential to comprehensively and systematically study and monitor food marketing to children on digital media and can also be leveraged to study food marketing in non-digital environments such as broadcast media and outdoor streetscapes. These systems can be part of ongoing efforts to enact and monitor adherence to comprehensive policies designed to reduce children’s exposure to unhealthy food marketing.

## Data Availability

Not applicable.

## Abbreviations

AI: Artificial intelligence
HFSS: High in fats, sugar and/or salt
WHO: World Health Organization
